# Syphilitic orchitis with long-term imaging follow-up and conservative treatment

**DOI:** 10.1016/j.eucr.2023.102447

**Published:** 2023-05-22

**Authors:** Shinya Miyazaki, Takashi Ueda, Ryosuke Tamai, Akihisa Ueno, Terukazu Nakamura

**Affiliations:** a Department of Urology, Saiseikai Suita Hospital, Suita, Osaka, Japan; b Department of Urology, Kyoto Prefectural University of Medicine, Kyoto City, Japan

## Abstract

An increased number of patients with syphilis was recently reported in Japan and the United States. Syphilitic orchitis, a late complication of syphilis, is a rare disease that presents as testicular swelling. In most cases, a diagnostic orchiectomy is performed because of the possibility of testicular cancer. We report a case treated conservatively with antibiotics considering the possibility of syphilitic orchitis based on the blood test results and describe long-term changes observed in the imaging findings.

## 1 Introduction

In Japan, there were 12,966 new syphilis cases in 2022 (103.2 per million population), a 1.6-fold increase from 2021.^1^ The number of syphilis patients continues to increase in the United States as well.^2^ Syphilitic orchitis, a late complication of syphilis, is a rare disease that presents as testicular swelling and must be differentiated from testicular cancers, which often present with similar symptoms. Although several cases have been reported in which inguinal orchiectomy was performed for both diagnosis and treatment and the condition diagnosed pathologically, there are few reports of the course of conservative treatment. Here we report a case of suspected syphilitic orchitis based on blood tests that was treated conservatively with antibiotics and subjected to long-term imaging follow-up for over 1 year.

## 2 Case presentation

**Fig. 1:**
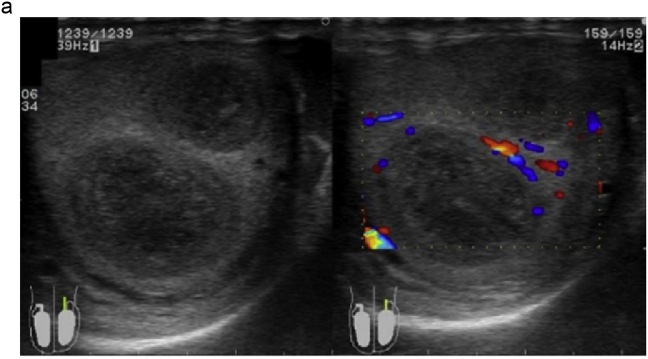

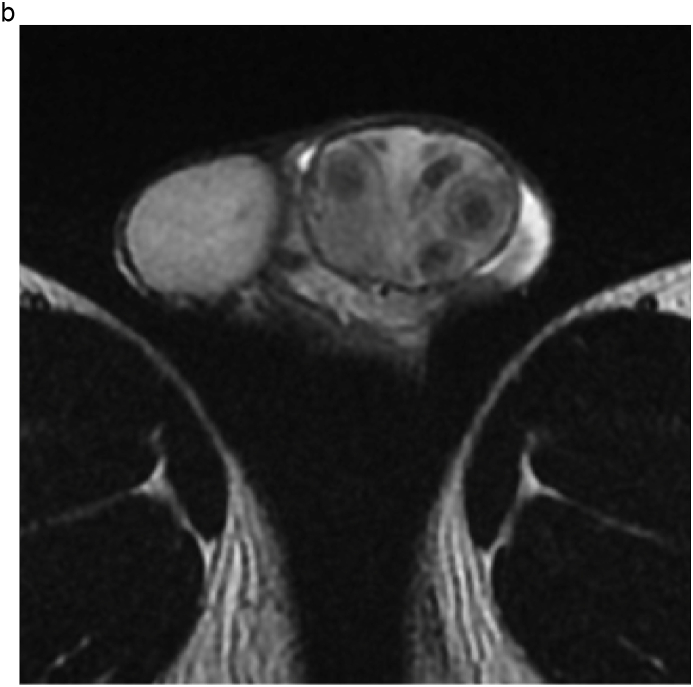

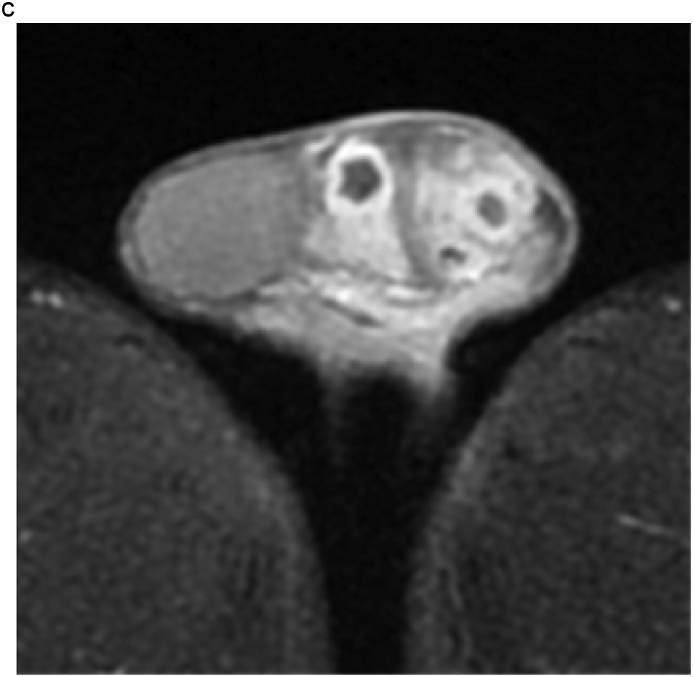
(a) Multiple hypoechoic regions and marginal Doppler signal enhancement are visible. (b) T2-weighted MRI showing multiple nodules with low signal intensity versus the normal testis. (c) In the early enhancement phase of T1-weighted MRI, marginal enhancement is prominent.

A 28-year-old man visited our institution with a chief complaint of swelling of the left testis and no fever. The patient had no history of sexual intercourse with sex workers. The testis was firm and mildly tender, and ultrasonography (US) showed multiple intratesticular masses and increased Doppler signals predominantly at their margins (Fig. 1a). All testicular tumor markers, including lactate dehydrogenase, α-fetoprotein, and β-human chorionic gonadotropin, were negative, and no increase in the inflammatory response was observed. No ulcers were observed on the skin, including on the penis. Magnetic resonance imaging (MRI) showed that the left testis was markedly swollen, with a major axis of 60 mm. Numerous nodules were observed inside the testis that were enhanced in a ring shape from the early phase (Fig. 1b and c). Computed tomography showed no evidence of metastasis, such as lymphadenopathy.

**Fig. 2:**
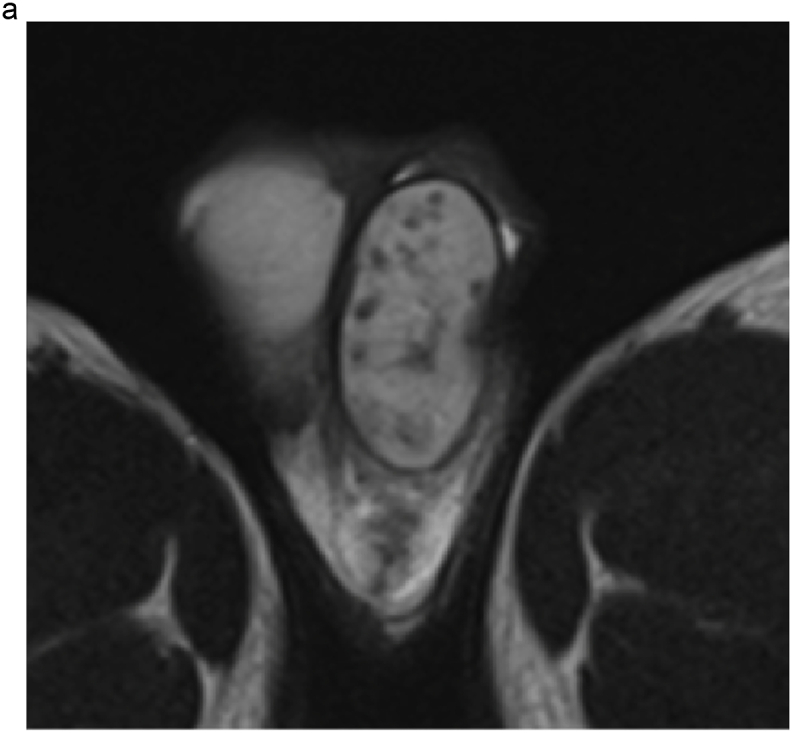

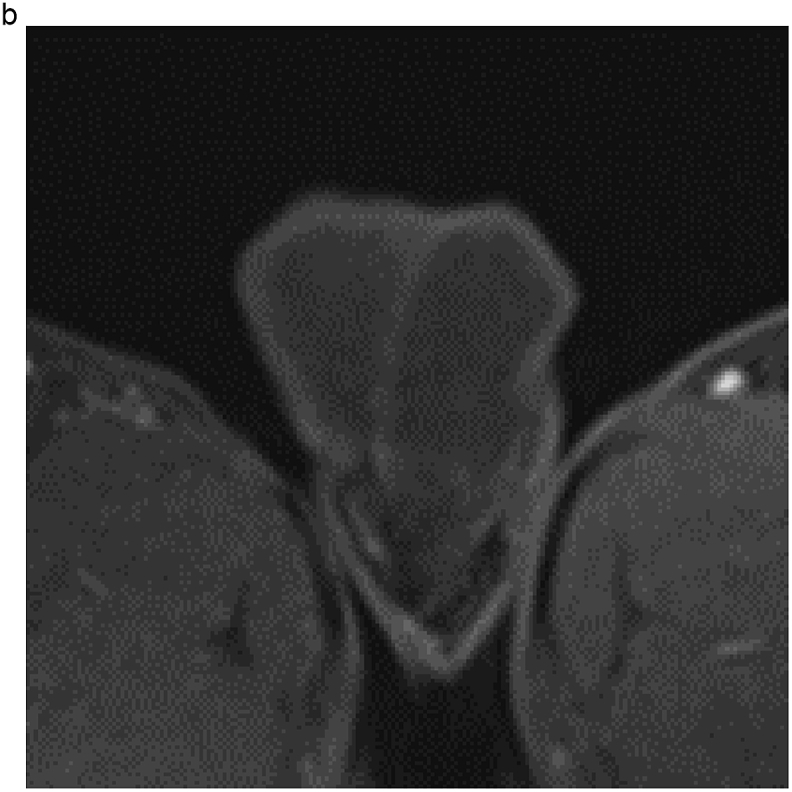

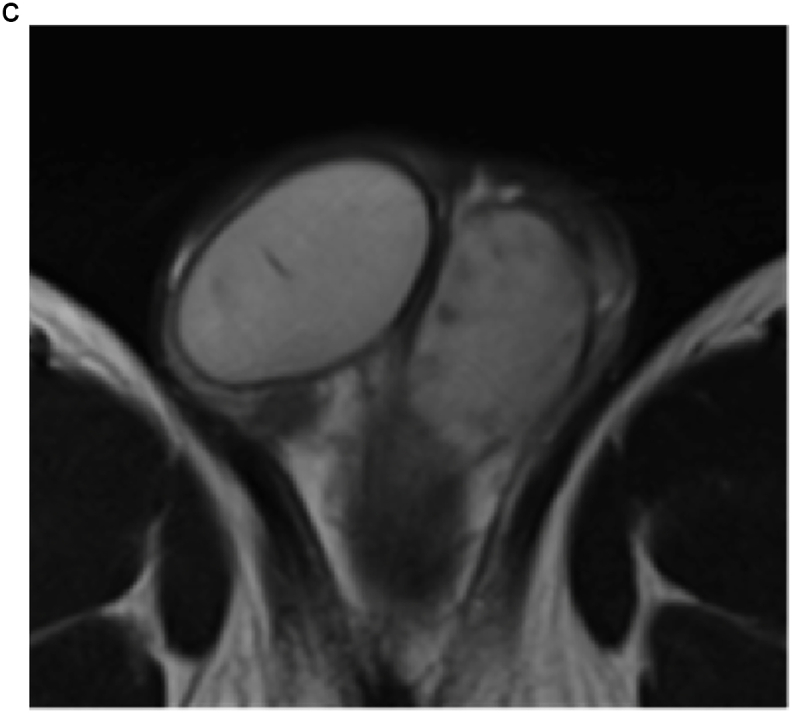

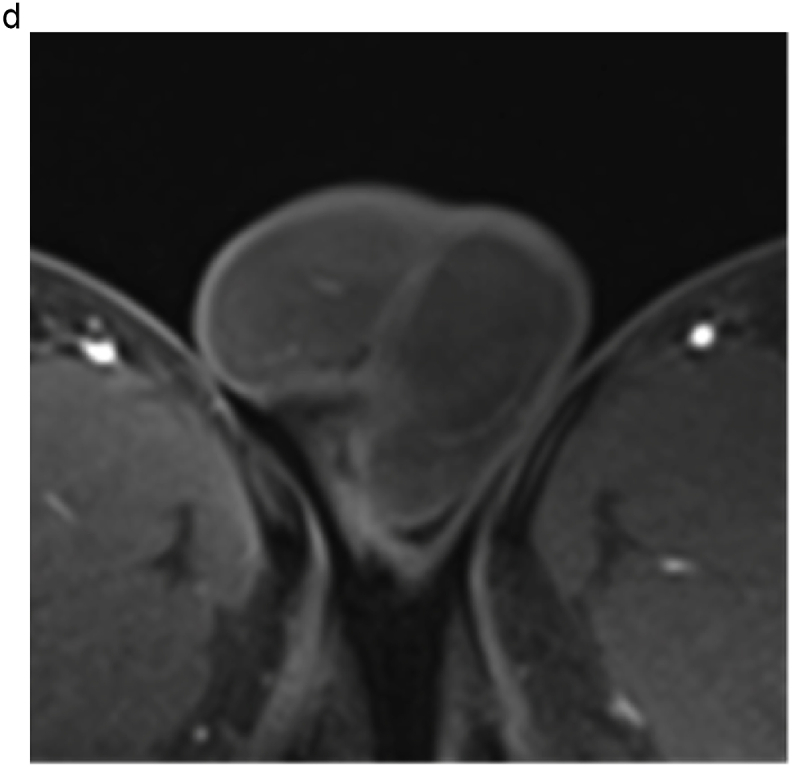

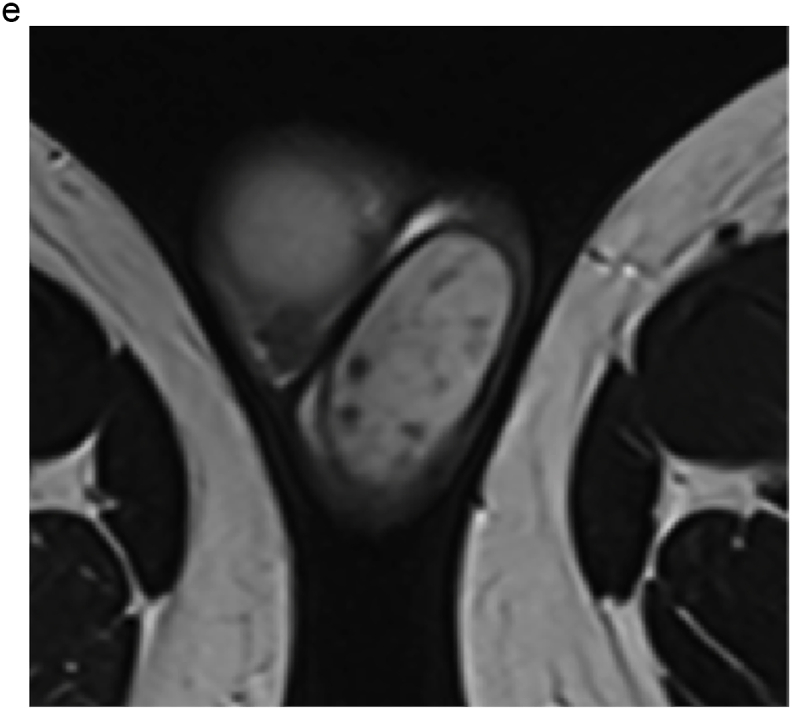

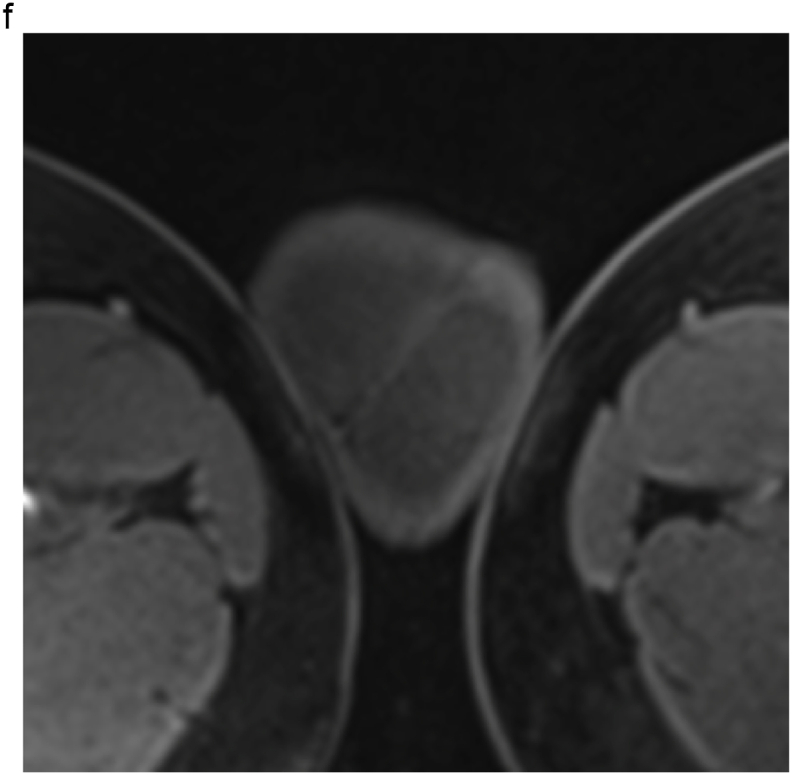
(a) T2-weighted MRI taken at 4 months after the initiation of conservative syphilis treatment showing that the mass had decreased in size. (b) At 4 months, the contrast enhancement had decreased markedly. (c) MRI taken at 10 months after the initiation of treatment. (d) At 10 months, the contrast enhancement remained decreased. (e) MRI taken at 16 months after the initiation of treatment. On T2-weighted images, the mass persisted. (f) At 16 months, no contrast enhancement was observed.

**Fig. 3:**
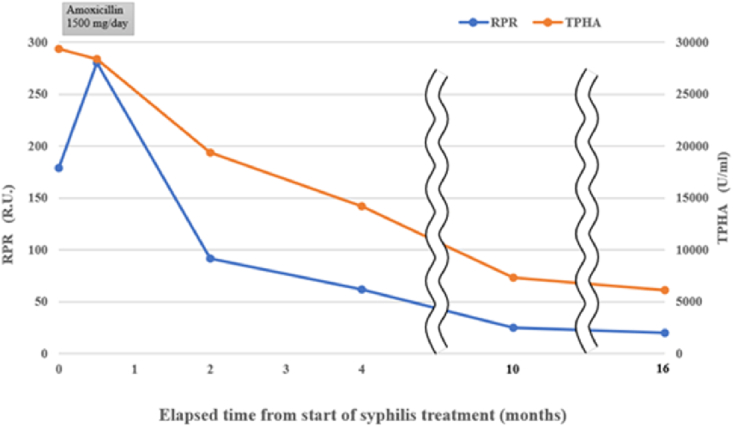
Graph of the patient's clinical course showing that the RPR and TPHA levels decreased after the start of antibiotic treatment.

Based on these results, the patient was scheduled to undergo an inguinal orchiectomy for suspected testicular cancer. However, preoperative blood tests were strongly positive for rapid plasma reagin (RPR) and *Treponema pallidum* hemagglutination assay (TPHA). Human immunodeficiency virus test results were negative for both antigens and antibodies. After we explained the possibility of testicular enlargement due to syphilis, the patient underwent testicular preservation. US and MRI follow-up examinations were performed after initiating syphilis treatment with oral amoxicillin. Amoxicillin (500 mg) was prescribed three times a day for 4 weeks. The RPR was sufficiently reduced, and syphilis was deemed cured. The testicular swelling and multiple nodules improved over time with the initiation of antibiotic therapy; however, the nodules persisted on MRI at 16 months (Fig. 2, Fig. 3).

## 3 Discussion

This case demonstrates that syphilitic orchitis can be treated conservatively with antibiotics. We also observed the healing process of associated testicular masses using US and MRI.

Teo et al. treated syphilitic orchitis conservatively with antibiotics and evaluated testicular lesions using US. After 10 months, the patient achieved complete remission.^3^ In our case, imaging follow-up was performed for 16 months from the start of treatment, but the nodule persisted. However, its enhancement was markedly decreased, indicating therapeutic efficacy.

MRI findings in syphilitic orchitis have been reported. Low signal intensity in T2-weighted images compared to normal testes was reported.^4^ On US, lesions are hypoechoic compared to normal tissue, and enhanced Doppler signals in the marginal area have been reported.^5^ In our case, the lesion showed low signal intensity on T2-weighted MRI with multiple hypoechoic areas and enhanced peripheral Doppler signals consistent with the above-mentioned reports.

Our case showed imaging findings of syphilitic orchitis that improved over time with antibiotic treatment. However, it was difficult to differentiate from testicular cancer based on clinical symptoms and imaging findings at the time of syphilis treatment initiation. Therefore, appropriate follow-up imaging is necessary when selecting conservative treatment. In the future, it will be necessary to examine how conservative treatment affects functional aspects such as sperm and hormone production in the long term. If no improvement in swelling is observed during treatment, it may be necessary to consider an orchiectomy.

## 4 Conclusion

The incidence of syphilitic orchitis is expected to increase in the future as the number of patients with syphilis increases. Conservative treatment with antibiotics should be considered when treating testicular enlargement in patients with syphilis while confirming imaging findings.

## Consent

Consent was obtained from the patient for the publication of this case report.

## Author contributions

Shinya Miyazaki: writing of the original draft. Takashi Ueda: writing, review, and editing. Ryosuke Tamai: data curation. Akihisa Ueno: data curation. Terukazu Nakamura: supervision.

## Funding

This research did not receive any specific grant from funding agencies in the public, commercial, or not-for-profit sectors.

## Declaration of competing interest

The authors declare no conflicts of interest.
